# Clinical practice guidelines and experts’ consensuses of traditional Chinese herbal medicine for novel coronavirus (COVID-19): protocol of a systematic review

**DOI:** 10.1186/s13643-020-01432-4

**Published:** 2020-08-03

**Authors:** Yuxi Li, Juan Li, Dongling Zhong, Yue Zhang, Yonggang Zhang, Yan Guo, Mike Clarke, Rongjiang Jin

**Affiliations:** 1 School of Acupuncture-Moxibustion and Tuina, Chengdu University of Traditional Chinese Medicine, Chengdu, China; 2 School of Health Preservation and Rehabilitation, Chengdu University of Traditional Chinese Medicine, Chengdu, Sichuan China; 3 West China Hospital, Sichuan University, Chengdu, China; 4 Xiyuan Hospital, China Academy of Traditional Chinese Medicine, Beijing, China; 5 Northern Ireland Clinical Trials Unit and Methodology Hub, Centre for Public Health, Queen’s University Belfast, Belfast, UK

**Keywords:** COVID-19, Coronavirus, Pneumonia, Traditional Chinese herbal medicine, Guideline, Systematic review

## Abstract

**Background:**

The World Health Organization declared on March 11, 2020, that the spread of the severe acute respiratory syndrome coronavirus 2 (SARS-Cov-2) has escalated from epidemic into pandemic. As the initial outbreak area, China has taken multiple active measures to deal with the epidemic. Updated versions of diagnosis and treatment guideline for novel coronavirus (COVID-19) patients have been issued, and traditional Chinese herbal medicine has been recommended as a treatment. The objective of this study will be to summarize the recommendations in current clinical practice guidelines about the use of traditional Chinese herbal medicine for COVID-19 patients. We will also evaluate and report on the methodological and reporting quality of these guidelines.

**Methods:**

In this systematic review, we will search for guidelines, expert consensuses, and policy documents published since December 2019 in electronic databases (e.g., PubMed, EMBASE, and Chinese databases) and on websites of governments or organizations (e.g., The National Guideline Clearinghouse [NGC], Guidelines International Network [GIN], National Institute for Health and Clinical Excellence (NICE), Scottish Intercollegiate Guidelines Network [SIGN], and WHO). Eligible documents will be independently selected, and relevant data will be independently extracted by two reviewers. We will also independently evaluate the methodological quality and reporting quality of the included guidelines, using the Appraisal of Guidelines for REsearch & Evaluation (AGREE) II tool and Reporting Items for Practice Guidelines in Healthcare (RIGHT) statement, respectively. Any discrepancies will be discussed and resolved through discussion among the reviewers. We will use the extracted information to summarize their recommendations for traditional Chinese herbal formulae and Chinese patent medicine for COVID-19 patients and to summarize the strength and quality of these recommendations with reference to the results of AGREE II and RIGHT tools.

**Discussion:**

This review will summarize the recommendations in current clinical practice guidelines and provide insight into the implementation strategies for traditional Chinese herbal medicine in COVID-19 patients.

**Systematic review registration:**

PROSPERO CRD42020179205

## Introduction

Novel coronavirus (COVID-19) pneumonia, caused by severe acute respiratory syndrome coronavirus 2 (SARS-CoV-2), was identified in Wuhan, China, in late December 2019 [1, 2]. COVID-19 patients mainly present with flu-like symptoms (such as fever, cough, and dyspnea) and some other systemic symptoms (such as malaise, fatigue, and diarrhea) [3, 4]. With a range of 1 to 14 days for the estimated incubation period and various severity of clinical symptoms [5, 6], human-to-human transmission of infectious disease has rapidly spread across the globe. The World Health Organization (WHO) declared a pandemic on March 11, 2020 [7]. With a global total now of more than 1 million cases, COVID-19 patients have been diagnosed in more than 200 countries [8].

As the initial outbreak area, China has taken multiple active measures to deal with COVID-19. The National Health Commission of China established the first version of Diagnosis and Treatment Guideline of Pneumonia Caused by Novel Coronavirus on January 16, 2020. This contained the etiology of COVID-19, clinical characteristics of patients, diagnosis, and treatments of this disease. As more information became available, updated versions of this diagnosis and treatment guideline were issued. Since the third version of National COVID-19 Diagnosis and Treatment Guideline, traditional Chinese herbal medicine has been recommended as a treatment for COVID-19, based on different stages and syndrome differentiations of disease [9].

Traditional Chinese herbal medicine has played an important role in thousand-of-years’ combat between Chinese doctors and pandemic and endemic diseases. During the outbreak of severe acute respiratory syndrome (SARS) coronavirus in the late 2002 in Guangdong, China, traditional Chinese herbal medicine showed beneficial effects such as relief of symptoms, decrease of mortality, and control of infection in patients with SARS [10–13]. With accumulated experiences of its anti-viral properties for the treatment of SARS and strong encouragement from the Chinese government [14, 15], traditional Chinese herbal medicine has also been applied in treating COVID-19. The State Council Information Office of China reported on March 23, 2020, that 74,187 COVID-19 patients (91.5% of total confirmed COVID-19 cases in China) had been treated with traditional Chinese herbal medicine with promising outcomes [16].

Along with the guideline issued by the National Health Commission of China, provinces, cities, and autonomous regions have also released clinical practice guidelines recommending traditional Chinese herbal medicine. The syndrome differentiations, treating principles, and recommended formulae may differ across these guidelines because of the different characteristics of their respective regions and population. Other reviews have been published which consider the treatment of COVID-19 patients with traditional Chinese herbal medicine and the guidelines for treating children with COVID-19 with herbal medicine [17, 18]. The objective of this systematic review is to systematically summarize the recommendations in current clinical practice guidelines about the use of traditional Chinese herbal medicine for COVID-19 patients. We will also evaluate and report on the methodological and reporting quality of these guidelines.

## Method

### Protocol registration and reporting

This systematic review is registered in the Prospective Register of Systematic Review (PROSPERO) database (CRD42020179205). This protocol is reported in accordance with the reporting guidance provided in the Preferred Reporting Items for Systematic Reviews and Meta-analyses Protocols (PRISMA-P) statement [19] (see checklist in Additional file 1).

### Eligibility criteria

#### Inclusion criteria

##### Study design

We will include clinical practice guidelines, experts’ consensus statements, and guidance documents (systematically developed statements to assist practitioners and patient decisions about appropriate healthcare for specific circumstances) published by any advising body or healthcare organization since December 2019, which provide information on the use of traditional Chinese herbal medicine therapy for COVID-19 patients.

##### Participants

Recommendations for patients diagnosed with COVID-19 pneumonia and the associated SARS-CoV-2 virus as confirmed by clinical tests (e.g., positive real-time fluorescent reverse transcription-polymerase chain reaction of respiratory or blood samples for SARS-CoV-2 virus nucleic acid, highly homologous of the respiratory specimen or blood specimen sequenced to SARS-CoV-2 virus). There will be no restrictions with respect to age, gender, or ethnicity.

##### Intervention

We will include recommendations for Chinese herbal medicine including Chinese medicinal formulae and Chinese patent medicine regardless of their forms (e.g., pill, powder, oral liquid). See Additional file 2 for definitions and examples of Chinese herbal medicine.

#### Exclusion criteria

We will exclude old versions of guidelines or consensus statement published by a single advising body or healthcare organization.

### Databases and search strategy

We will search for guidelines, expert consensuses and policy documents published since December 2019 in the following electronic databases: PubMed, Embase, Chinese Biomedical Literature Database (CBM), China National Knowledge Infrastructure (CNKI), Chinese Science and Technology Periodical Database (VIP), and Wanfang database (Wanfang Data). In addition, we will search other sources of guidelines including the following: The National Guideline Clearinghouse (NGC), Guidelines International Network (GIN), National Institute for Health and Clinical Excellence (NICE), Scottish Intercollegiate Guidelines Network (SIGN), and WHO. We will use search strategies that combine terms relating to novel coronavirus, traditional Chinese medicine, and guideline. The search strategy for PubMed is described in Additional file 3, and suitably modified search strategies will be applied to the other electronic databases. We will also search grey literature via websites of governments or organizations. No language restrictions will be applied.

### Study selection

We will upload search results into the EndNote X9 reference management software. Duplicated references will be filtered and removed. Two independent reviewers (YXL and DLZ) will screen titles and abstracts to identify potentially eligible documents, which will be retrieved in full text for final review. Any disagreements will be discussed and arbitrated by a third reviewer (YGZ).

### Data extraction

We will design a data extraction form based on characteristics of included guidelines and consensuses. In order to achieve a consistency (at least 80%) in extracted items, we will pilot test the data extraction form with data extractors using a sample of eligible documents. Results of this pilot extraction will be discussed among review authors and extractors before finalizing it. Data extraction will be conducted independently by two reviewers (JL and YZ) and will include the following data: (1) characteristics of guidelines: name, type, source, and date; (2) stage of disease; (3) syndrome differentiation; (4) treatment principle; (5) recommended formula; and (6) recommended Chinese patent medicine etc. Disagreements will be resolved by discussion among the review team.

### Quality assessment

We will evaluate the methodological quality and reporting quality of the included guidelines using the Appraisal of Guidelines for REsearch & Evaluation (AGREE) II tool [20–22] and Reporting Items for Practice Guidelines in Healthcare (RIGHT) statement, respectively. In order to achieve consistency of at least 80% in the quality assessments, the assessors will pre-assess a sample of eligible documents and discuss their assessment among review authors and assessors. After that, two independent reviewers (YXL and JL) will appraise the quality of included guidelines. Discrepancies will be discussed and resolved among the reviewers.

### Data synthesis

With the information extracted from the included guidelines, we will summarize the recommendations made for traditional Chinese herbal formulae and Chinese patent medicine for COVID-19 patients. The recommendations of prescriptions for different stages and syndrome differentiations of COVID-19 will also be summarized. We will present the evidence in narrative format and tables with descriptive statistics (e.g., frequencies). The assessments from the use of the AGREE II and RIGHT tools will be presented in tables with reporting rate of each item and the overall reporting rate. These findings will be discussed in light of the strength and quality of these recommendations with reference to the results of AGREE II and RIGHT tools.

## Discussion

This systematic review will comprehensively congregate information from and appraise the quality of guideline recommendations for traditional Chinese herbal medicine for COVID-19 patients. Other reviews have either examined the application of traditional Chinese herbal medicine for COVID-19 patients or investigated current management for COVID-19 patients. Examples of other studies include Ho and colleagues’ work on the case definition, clinical classifications, and relevant treatments of COVID-19 patients, in accordance to recommendations of the 7th edition of the National COVID-19 Diagnostic and Treatment Guideline of China [23]. In addition, Du and Luo have summarized the theoretical foundation and potential effect of Chinese herbs on COVID-19 patients [24, 25] and Yang and colleagues outlined clinical data for SARS-CoV and compared the evidence of current applications of traditional Chinese herbs in the treatment of COVID-19 patients [26].

Given the many guidelines and consensuses that are now being regularly updated to advise clinical practices on the use of traditional Chinese herbal medicine for COVID-19 patients, this review will provide an important overall summary of their recommendations and provide insight into relevant implementation strategies.

There may be some limitations in our retrieving and reviewing of the evidence: (1) we will only search Chinese and English databases, so we may miss guidelines published in other languages; (2) COVID-19 is a very urgent public health issue requiring rapid responses, which means that the production of associated guidelines might not follow the standards for the development of guidelines in more usual times. Some other considerations are that according to traditional Chinese medicine, Chinese herbs should be prescribed based on different constitutions of the population and the environment, and we will classify the data and discuss the results based on the theory. If changes are needed for this protocol, we will update our PROSPERO entry as necessary. We intend to publish this review in a peer-reviewed journal.

## Supplementary information

**Additional file 1.** PRISMA-P 2015 Checklist.

**Additional file 2.** Definition and examples of Chinese herbal medicine.

**Additional file 3.** Search Strategy for PubMed.

## Data Availability

Not applicable.

## Abbreviations

COVID-19: The novel coronavirus
SARS-CoV-2: Severe acute respiratory syndrome coronavirus 2
SARS: Severe acute respiratory syndrome
WHO: World Health Organization
PROSPERO: Prospective Register of Systematic Reviews
PRISMA-P: Preferred Reporting Item for Systematic Review and Meta-analysis Protocol
CBM: Chinese Biomedical Literature Database
CNKI: China National Knowledge Infrastructure
VIP: Chinese Science and Technology Periodical Database
NGC: The National Guideline Clearinghouse
GIN: Guidelines International Network
NICE: National Institute for Health and Clinical Excellence
SIGN: Scottish Intercollegiate Guidelines Network
AGREE: The Appraisal of Guidelines for REsearch & Evaluation
RIGHT: Reporting Items for Practice Guidelines in Healthcare
