# Supplementary material for: Clinical practice guidelines and experts’ consensuses of traditional Chinese herbal medicine for novel coronavirus (COVID-19): protocol of a systematic review
Source: Syst Rev. 2020 Aug 3;9:170. doi: 10.1186/s13643-020-01432-4 (PMC7397967; doi:10.1186/s13643-020-01432-4)
Supplement: Supplementary file 2 — Additional file 2. Definition and examples of Chinese herbal medicine. [file 13643_2020_1432_MOESM2_ESM.docx]

**Additional file 2 Definition and examples of Chinese herbal medicine**

| **Category** | **Definition** | **Examples** |
| --- | --- | --- |
| Chinese medicinal formulae | Prescription of traditional Chinese herbal medicine composed of several herbs according to compatibility principle in traditional Chinese medicine and summarized clinical experience. | Qingfei Paidu decoction, Maxing Shigan decoction |
| Chinese patent medicine | A kind of traditional Chinese medicine with traditional Chinese medicine as the raw material. Under the guidance of the theory of traditional Chinese medicine, it is processed into a certain dosage form of Chinese herbs according to the prescribed prescription and preparation technology. | Lianhua Qingwen granules, Huoxiang Zhengqi liquid |
