# Supplementary material for: Clinical practice guidelines and experts’ consensuses of traditional Chinese herbal medicine for novel coronavirus (COVID-19): protocol of a systematic review
Source: Syst Rev. 2020 Aug 3;9:170. doi: 10.1186/s13643-020-01432-4 (PMC7397967; doi:10.1186/s13643-020-01432-4)
Supplement: Supplementary file 3 — Additional file 3. Search Strategy for PubMed. [file 13643_2020_1432_MOESM3_ESM.docx]

**Additional file 3 Search Strategy for PubMed**

| **#1** | Search novel coronavirus[Title/Abstract] |
| --- | --- |
| **#2** | Search COVID-19[Title/Abstract] |
| **#3** | Search 2019-nCoV[Title/Abstract] |
| **#3** | Search novel coronavirus pneumonia[Title/Abstract] |
| **#4** | Search COVID-19 pneumonia[Title/Abstract] |
| **#5** | Search 2019-nCoV pneumonia[Title/Abstract] |
| **#6** | Search #1 OR #2 OR #3 OR #4 OR #5 |
| **#7** | Search traditional Chinese medicine[MeSH Terms] |
| **#8** | Search Drugs, Chinese Herbal[MeSH Terms] |
| **#9** | Search traditional Chinese herbal medicine[Title/Abstract] |
| **#10** | Search Chinese herb*[Title/Abstract] |
| **#11** | Search #7 OR #8 OR #9 OR #10 |
| **#12** | Search (guideline OR practice guideline OR consensus development conference RO consensus OR standards OR recommendation)[Title/Abstract] |
| **#13** | Search #6 AND #11 AND #12 |
